# Sustaining Attention for a Prolonged Duration Affects Dynamic Organizations of Frequency-Specific Functional Connectivity

**DOI:** 10.1007/s10548-020-00795-0

**Published:** 2020-09-14

**Authors:** Jia Liu, Yongjie Zhu, Hongjin Sun, Tapani Ristaniemi, Fengyu Cong

**Affiliations:** 1 School of Biomedical Engineering, Faculty of Electronic Information and Electrical Engineering, Dalian University of Technology, Dalian, 116024 China; 2 Faculty of Information Technology, University of Jyvaskyla, 40014 Jyvaskyla, Finland; 3 Department of Psychology, Neuroscience and Behavior, MacMaster University, Hamilton, ON L8S4K1 Canada; 4 School of Artificial Intelligence, Faculty of Electronic Information and Electrical Engineering, Dalian University of Technology, Dalian, 116024 China; 5 Key Laboratory of Integrated Circuit and Biomedical Electronic System, Dalian University of Technology, Dalian, 116024 China

**Keywords:** Sustained attention, Vigilance decrement, Motivation, Frequency-specific dynamic functional connectivity, Weighted phase lag index, Tensor component analysis

## Abstract

Sustained attention encompasses a cascade of fundamental functions. The human ability to implement a sustained attention task is supported by brain networks that dynamically formed and dissolved through oscillatory synchronization. The decrement of vigilance induced by prolonged task engagement affects sustained attention. However, little is known about which stage or combinations are affected by vigilance decrement. Here, we applied an analysis framework composed of weighted phase lag index (wPLI) and tensor component analysis (TCA) to an EEG dataset collected during 80 min sustained attention task to examine the electrophysiological basis of such effect. We aimed to characterize the phase-coupling networks to untangle different phases involved in sustained attention and study how they are modulated by vigilance decrement. We computed the time–frequency domain wPLI from each block and subject and constructed a fourth-order tensor, containing the time, frequency, functional connectivity (FC), and blocks × subjects. This tensor was subjected to the TCA to identify the interacted and low-dimensional components representing the frequency-specific dynamic FC (fdFC). We extracted four types of neuromakers during a sustained attention task, namely the pre-stimulus alpha right-lateralized parieto-occipital FC, the post-stimulus theta fronto-parieto-occipital FC, delta fronto-parieto-occipital FC, and beta right/left sensorimotor FCs. All these fdFCs were impaired by vigilance decrement. These fdFCs, except for the beta left sensorimotor network, were restored by rewards, although the restoration by reward in the beta right sensorimotor network was transient. These findings provide implications for dissociable effects of vigilance decrement on sustained attention by utilizing the tensor-based framework.

**Electronic supplementary material:**

The online version of this article (10.1007/s10548-020-00795-0) contains supplementary material, which is available to authorized users.

## Introduction

Human attentional resources are not limitless. Sustaining attention on stimuli for a prolonged duration results in task performance declines and mental fatigue increases. This effect is known as time-on-task effect or vigilance decrement (Davies and Parasurman 1982; Gillberg and Åkerstedt 1998; Lim and Dinges 2008; Mackworth 1948; See et al. 1995). Vigilance decrement leads to increased safety risks and decreased productivity at work. Efforts have been made to explore the mechanisms of vigilance decrement. In particular, three theoretical categories—underload, overload, and motivational control—have emerged in the mechanisms of vigilance decrement (Liu et al. 2020a; Reteig et al. 2019). The underload theories maintain that cognitive tasks are too monotonous to maintain task performance for a prolonged period of time (Manly et al. 1999). Whereas the overload theories hold that a limited pool of cognitive resources is depleted during a long period of task performance (Caggiano and Parasuraman 2004). Furthermore, the motivational control theories insist that the decrement of vigilance is associated with mental representations of costs and benefits and the task performance decreases when the costs outweigh the benefits (Kurzban et al. 2013). However, these three theories still have limitations to interpret all fatigue-related changes. In recent years there have been theoretical frameworks synthesizing different theoretical categories. For instance, Boksem and colleagues (Boksem and Tops 2008) proposed a hybrid model synthesizing the motivational control and energetical costs, stating that human task performance is determined by the energetical state and the mental representations of costs and benefits. Other hybrid models synthesizing underload and overload theories (Thomson et al. 2015) and synthesizing underload and motivational control theories (Seli et al. 2015) have also been proposed in the literature. Despite substantial efforts have been made for this, the mechanisms of vigilance decrement are still ambiguous.

Sustained attention has been widely used in the studies of vigilance decrement in the laboratory because tests of sustained attention are reliable and the neural mechanisms of sustained attention have been fairly well acknowledged. Sustained attention studies using perfusion functional magnetic resonance imaging (fMRI) and fMRI have uncovered that the fronto-parietal attention network decreases during prolonged sustained attention task engagement (Lim et al. 2010; Taya et al. 2018). Previous electroencephalogram (EEG) work of sustained attention has implicated that the theta and alpha frequency bands mainly at frontal and parietal brain regions are associated with vigilance decrement (Sauseng et al. 2007; Sun et al. 2014). While the summarized attention network and oscillations are useful neuromakers of vigilance decrement, few studies have addressed the frequency-specific dynamic functional connectivity (fdFC) without a prior selection of time windows, frequency bands or brain regions in the functional connectivity (FC) or oscillatory analysis.

In essence, whole-brain interactions through phase synchronization in specific frequency band form and dissolve dynamically and transiently to support cognitive processes (Bola and Sabel 2015; Fries and Str 2015; O’Neill et al. 2017; Rosenberg et al. 2016; Vidaurre et al. 2018). Sustained attention encompasses a variety of fundamental cognitive processes, including attentional preparatory, attentional stability, working memory, and enhancement or inhibition of selected or unselected information (Clark et al. 2015; Reteig et al. 2019; Rosenberg et al. 2016; Slagter et al. 2016). Brain regions rapidly shift the patterns of FC on the basis of the cognitive process demands (Cole et al. 2013). To successfully execute a sustained attention task, the fdFCs should emerge dynamically, with the temporal scale of milliseconds (Bola and Sabel 2015). Nevertheless, it is still unclear how oscillations are involved in brain networks during a sustained attention task. Little is known which stage or a combination of stages are impaired by vigilance decrement.

High-temporal resolution modality matching the rapid timescales of the brain is efficient for tracking the dynamics of FC. In the present study, we adopt a high-temporal resolution EEG dataset collected when participants performed a sustained attention task as long as 80 min and they were provided with unexpected monetary rewards 20 min before the end of the task (Reteig et al. 2019; Slagter et al. 2016). A different set of results based on this dataset extracted three univariate neuromarkers of vigilance decrement, consisting of the pre-stimulus alpha power, the early post-stimulus P1/N1 component, and the post-stimulus theta phase (Reteig et al. 2019). However, they did not use multivariate fashion through the integrity of the whole-brain networks. By utilizing the analysis framework composed of the weighted phase lag index (wPLI) and tensor component analysis (TCA), we aim to characterize the fdFC corresponding to temporal-spectral-spatial signatures that can be used to interpret the neural mechanisms of different phases of sustained attention and to reflect the modulations by vigilance decrement.

The wPLI is used to estimate the contributions of phase leads and lags, with the advantage of being insensitive to the volume-conduction or noise (Vinck et al. 2011). The TCA is applied to characterize the interacted and low-dimensional components. Compared with the matrix decomposition analysis, the TCA provides a good approach for identifying brain activities in multiple domains simultaneously without stacking or concatenating the data (Cong et al. 2015,2014; Liu et al. 2020b). The analysis framework was firstly proposed by our team and successfully derived the temporal, spectral, and spatial modes of covariation (third-order tensor) during freely listening to music (Zhu et al. 2019). The reliability and stability of this analysis framework (third-order tensor) was further validated using the MEG data collected during a hand movement task and a working memory task (Zhu et al. 2020a). We then apply this framework to track the temporal, spectral, spatial, and feature modes of covariation (fourth-order tensor) simultaneously during a prolonged sustained attention task. In order to find the divergences between different responses during sustained attention, we perform the same framework in conditions of correct rejections, hits, and misses, respectively. We compute the time–frequency domain wPLI between whole-brain electrodes from each block and subject. Considering the equalization of the trial numbers, we perform the wPLI for 100 times and then average the time–frequency domain FC. We construct a fourth-order tensor including time points, frequency bins, pairs of connections, and subjects × blocks. The fourth order tensor is subjected to the TCA to derive the interacted and low-dimensional TCA components, consisting of the temporal factor (dynamic temporal fluctuations), spectral factor (oscillatory distributions), connectivity factor (FC), and features (representations of fdFC influenced by time-on-task and motivation). With the use of the tensor-based framework, we seek to identify which frequency bands, in which time windows, and how the FC patterns involved in cognitive tasks and provide evidence for the modulations by complex factors.

## Materials and Methods

### Data Description

We adopted a sustained attention EEG dataset published on the Open Science Framework (OSF) platform, which is a free, open platform to support our research and enable collaboration. The contributors of this dataset have reported a different set of results in a prior study (Reteig et al. 2019). The description of the data and preprocessing procedure in detail could be found at the link: https://doi.org/10.17605/OSF.IO/EMF9H.

**Fig. 1:**
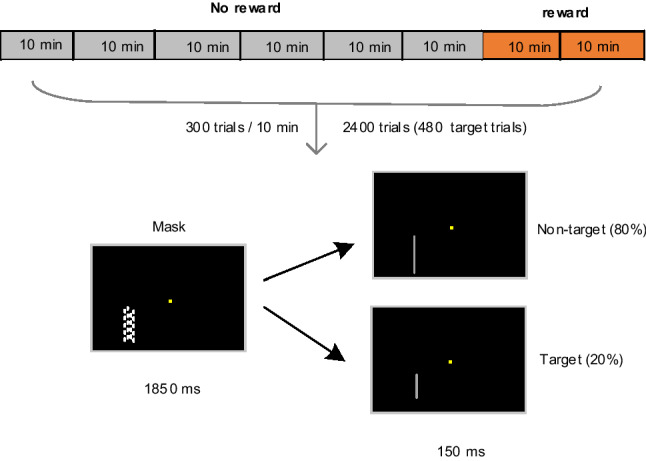
Outline of the experiment procedure and an example of one trial in the sustained attention task

The EEG data of twenty-one participants (ten males, aged 21.6 ± 3.4 years) collected during a modified version of sustained attention task (Maclean et al. 2009) was reported on the OSF platform. All stimuli were presented on a 17–inch monitor at a viewing distance of 100 cm. Participants were asked to maintain their fixation on a central yellow square (0.11° × 0.11°) against a black background throughout the task and covertly and continuously direct their attention to the stimuli located at 3° to the left and 1.5° lower than the fixation. The stimuli were presented only in the left hemifield, with the right hemifield never relevant. The outline of each trial is presented in Fig. 1. In each trial of 2 s, a light gray line was shortly presented at the to-be-attended location for 150 ms and was followed by a mask stimulus for the remaining 1850 ms. The light gray line with a width of 0.03° could either be a long (non-target, 80%) or short line (target, 20%). The long line was fixed on 1.89° in length, whereas the short one was calibrated individually before the main task. Participants were instructed to conduct a response to the rare target with their right index figure and withhold a response to the non-target. The mask stimulus was composed of many lines (0.03° × 0.12°), positioned with a space of 0.21° × 2.44°. These lines were randomly shifted in a small height (within ± 0.06°) on each presentation to prevent participants from recognizing the length of (non-)target line relative to the mask lines.

The Parameter Estimation by Sequential Testing (PEST) (Maclean et al. 2009; Taylor and Creelman 1967) was adopted to adjust the length of the short (target) line for individual participants, achieving a minimum accuracy of 80% in the task. The short line length ranged from 1.21° to 1.59° (1.40° ± 0.01°). After the execution of PEST (7–13 min), participants performed the main task for an interval of 80 min, consisting of 2400 trials (480 target trials) in total (Fig. 1). At the beginning of the task and every 10 min (300 trials), participants were provided with two 7–point scales to evaluate their levels of motivation (1: “not motivated”, 7: “highly motivated”) and aversion (1: “no aversion”, 7: “strong aversion”). In the last 20 min of the main task, participants were informed an additional monetary reward of €30 (unknown to them until then) if they outperformed 65% of the other participants (Lorist et al. 2009). The instruction of monetary rewards—appeared at 60 min task-onset—disappeared until a button click or until a maximum of 60 s.

### Data Acquisition and Preprocessing

EEG data was recorded using the BioSemi ActiveTwo with 64 Ag/AgCl electrodes arranged according to the international 10–10 system. The EEG signals were digitized at a sampling rate of 512 Hz. Each electrode was referenced to a common mode sense electrode online. Two additional channels were placed to the left and the right earlobes and four other external electrodes were used to record the horizontal (left and right outer canthi) and vertical (below and above the left eye) EOGs.

The preprocessing was conducted in MATLAB with the EEGLAB toolbox (Delorme and Makeig 2004). The EEG signals were high-pass filtered at 0.1 Hz and then segmented into epochs from − 2000 to 3000 ms peri-stimulus with buffer zones to reconcile the edge effects. Bad channels were interpolated using the spherical spline interpolation. With a visual inspection, parts of epochs containing eye movements, muscle activities, and other artifacts were removed. By running the independent component analysis (ICA), artificial components distinguishable from the neural activities were removed. Epochs were average referenced and segmented into − 1000 to 1000 ms peri-stimulus. Based on the markers of stimuli and responses, epochs were divided into four conditions, namely correct rejections, false alarms, hits, and misses. The condition of false alarms was not analyzed because the number of trials was too small.

### Data Processing

**Fig. 2:**
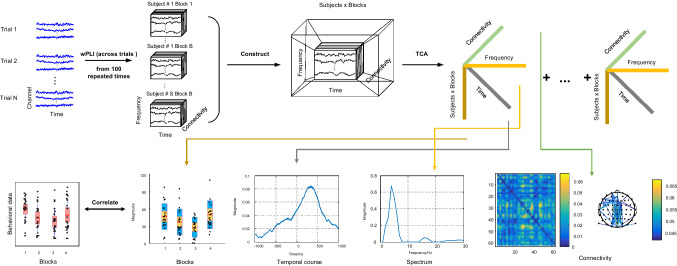
The pipeline of data processing. The number of stimulus-locked trials were equalized over blocks for each participant. The subsampling process was repeated for 100 times. For each time, the signals from all trials were decomposed with the complex Morlet wavelet and then calculated with the wPLI, generating a third-order tensor (Time by frequency by connectivity) for each block and participant. An average third-order tensor was obtained by averaging all third-order tensors from 100 repeated times. Based on the average tensor of each block and participant, we constructed a fourth-order tensor by concatenating the blocks and subjects together. The fourth-order tensor was subjected to the TCA to extract the demixed components containing temporal course, spectrum, connectivity, and representations of blocks and subjects (features). The related components involved in the sustained attention were selected based on prior knowledge in the literature and the significant correlations with behavioral data

The segmented epochs were further analyzed following the main steps of data processing. A schematic of the data analysis is demonstrated in Fig. 2.

#### Trial Binning

Consistent with previous analysis (Reteig et al. 2019), trials were split into eight 10–min blocks in the condition of correct rejections and binned into four 20–min blocks in conditions of hits and misses as the number of trials was too small. Obviously, the number of trials were different in each block for each participant, which might have a significant effect on results, especially the phase-based analysis results (Cohen 2014). Therefore, the number of trials was equalized across blocks for each participant by randomly selecting a minimum number of trials over blocks. Using the subsampling process, the number of trials in the correct rejections condition (167 ± 22.5, range = 124–213) and in the hits and misses conditions (24 ± 5.4, range = 11–33) was determined. The subsampling process was repeated 100 times. We computed the wPLI connectivity (Dynamic functional connectivity analysis section) at each time. The wPLI measures of all 100 times were further averaged to achieve the final value.

#### Dynamic Functional Connectivity Analysis

##### Time–Frequency Representations

The spectral densities were estimated from each trial using the continuous wavelet transform with the complex Morlet wavelets. The frequency band from 1 to 30 Hz was linearly spaced in a resolution of 1 Hz. To preserve the temporal precision in low and high frequency bands, the number of wavelet cycles was adjusted from 2 to 11. A total of ${n}_{f}=30$ linearly spaced frequencies and ${n}_{t}=$ 1024 time points (− 1000 to 1000 ms peri-stimulus) were estimated. Thus, we derived the time frequency representations ${S}_{c}^{n}\left(t,f\right)$ in time point $t\in [1,{n}_{t}]$ and frequency bin $f\in [1,{n}_{f}]$ for trial $n$, where $n\in [1,N]$, $N$ is the number of trials, $c\in [1,{n}_{c}]$, ${n}_{c}$ is the number of channels, ${n}_{c}=63$ after removing the on-line reference channel.

##### Weighted Phase Lag Index

The wPLI was used to quantify phase differences by the magnitude of imaginary of the cross-spectrum (Vinck et al. 2011). Compared with the phase lag index (PLI) (Stam et al. 2007), the wPLI is less sensitivity to noise and volume conduction because of the contribution of weighted phase leads and lags. We computed wPLIs between all pairs of channels in each time point and frequency bin:

1 $${wPLI }_{\left(c1, c2\right)}(t, f)=\frac{\left|{\sum }_{n=1}^{N}im\left({S}_{c1}^{n}\left(t,f\right){S}_{c2}^{n*}\left(t,f\right)\right)\right|}{{\sum }_{n=1}^{N}\left|im\left({S}_{c1}^{n}\left(t,f\right){S}_{c2}^{n*}\left(t,f\right)\right)\right|}$$

where ${S}_{c1}^{n}\left(t,f\right)$ and ${S}_{c2}^{n}\left(t,f\right)$ are time–frequency representations from two different channels $c1, c2\in [1,{n}_{c}]$ in time point $t\in [1,{n}_{t}]$ and frequency bin $f\in [1,{n}_{f}]$ at trial $n\in [1,N]$. $im\left(\right)$ represents the imaginary part of a complex value. $*$ is the complex conjugate and | | is an absolute operation. We then constructed a third-order tensor $\mathcal{P}$ with the dimensions of ${n}_{t}\times {n}_{f}\times C$ in each block and participant, where $C=1953$ denotes the number of pairs of channels $(63\times (63-1)/2)$. We computed the wPLI for 100 repeated times (Trial binning section) and averaged these 100 third-order tensors forming a final third-order tenor in each block and subject. In view of the blocks and subjects, we created a fifth-order tensor $\mathcal{O}$ with dimension of ${n}_{t}\times {n}_{f}\times C\times S\times B$, where $S=21$ is the number of participants and $B$ is the block amount, $B=8$ in the correct rejections condition and $B=4$ in the hits and misses conditions. Finally, we reshaped the tensor $\mathcal{O}$ into fourth-order tensor $\mathcal{X} \left({n}_{t}\times {n}_{f}\times C\times M\right)$ by concatenating the blocks and participants together, where $M=S\times B$.

#### Tensor Component Analysis

In general, the multi-mode data were stacked or concatenated to facilitate two-way processing methods (e.g., independent component analysis (ICA) and principal component analysis (PCA)) for extracting interested brain activities (Bernat et al. 2005; Cong et al. 2010; Dien 2010; Tenke and Kayser 2005; Vigário and Oja 2008; Zhu et al. 2020b). The procedures of stacking and concatenating inevitably lost potential interaction information (Cong et al. [Bibr CR18][Bibr CR16]). The TCA can be directly applied to the multi-way data, exploiting the interacted information among multiple modes (Hitchcock 1927). As one of the most fundamental models of TCA, the canonical polyadic (CP) model (A.Harshman 1970; Hitchcock 1927) was applied to extract demixed components in our study. As all elements in the fourth-order tensor $\mathcal{X}\in {\mathbb{R}}_{+}^{{n}_{t}\times {n}_{f}\times \mathrm{C}\times M}$ were nonnegative, the nonnegative constraint was used in the canonical polyadic decomposition (CPD) (Cichocki et al. 2009). For the input $\mathcal{X}$, the CPD is defined as an approximation of the sum of the outer products:

2 $${\mathbf{\mathcal{X}}} \approx \mathop \sum \limits_{j = 1}^{J} {\mathbf{\mathcal{X}}}_{j} = \mathop \sum \limits_{j = 1}^{J} a_{j} \circ b_{j} \circ c_{j} \circ d_{j}$$

where ${\mathcal{X}}_{j}$ is the component $j$ $\in \left[1,J\right]$ of $\mathcal{X}$, and $J$ is the number of TCA components. The outer product of four factor-vectors ${{\varvec{a}}}_{j}\circ {{\varvec{b}}}_{j}\circ {{\varvec{c}}}_{j}\circ {{\varvec{d}}}_{j}$ produces the rank-one tensor${\mathcal{X}}_{j}$. The operator $\circ$ is the outer product of the factor-vectors. In this application (Fig. 2), ${{\varvec{a}}}_{j}$ is the temporal factor illustrating the temporal fluctuations, and ${{\varvec{b}}}_{j}$ is the spectral factor characterizing the involvement of specific frequency band, and ${{\varvec{c}}}_{j}$ is the connectivity factor representing whole-brain FC, and ${{\varvec{d}}}_{j}$ is the feature factor indicating the alterations of specific time points, frequency bins, and FC affected by vigilance decrement and motivation.

The realization of CPD is to solve the following minimization problem:

3 $$_{{\varvec{A,B,C,D}}}^{{\min }} \frac{1}{2}\left\| {{\mathcal{X}} - \sum\limits_{{j = 1}}^{J} {{\varvec{a}}_{j} \circ {\varvec{b}}_{j} \circ {\varvec{c}}_{j} \circ {\varvec{d}}_{j} } } \right\|_{F}^{2}$$

where ${\varvec{A}} = [\user2{a}_{1} ,\user2{a}_{2} , \ldots ,\user2{a}_{j} ]$,${\varvec{B}} = [\user2{b}_{1} ,\user2{b}_{2} , \ldots ,\user2{b}_{j} ]$,${\varvec{C}} = [\user2{c}_{1} ,\user2{c}_{2} , \ldots ,\user2{c}_{j} ]$, and ${\varvec{D}} = [\user2{d}_{1} ,\user2{d}_{2} , \ldots ,\user2{d}_{j} ]$ are factor matrixes of the temporal course, spectrum, FC, and features. The operator ${\Vert \Vert }_{F}$ is the Frobenius norm. The minimization problem in Eq. (3) can be solved by iterative optimization methods. The hierarchical alternating least squares (HALS) was applied in our study because the validity and high performance of HALS have been confirmed by extensive studies (Cichocki et al. 2009, 2008, 2007). The component number $J$ was determined by the difference of fits (DIFFIT) (Cong et al. 2014, 2013b). The DIFFIT measures the differences in data fitting and is obtained by relative error and the explained sum of squares (Mørupa and Hansena 2009). The number of component $J$ was chosen from 1 to 40 and the data fitting was averaged across 10 repetitions of CPD. In theoretical, the optimal component number $J$ corresponds to the local maximum value of DIFFIT and a high data fit value.

#### Selection of TCA Components Modulated by Sustained Attention Tasks

By using the DIFFT, we determined $J$ TCA components containing temporal course, spectrum, FC, and variations of blocks over subjects. Here, we aimed to select the related components modulated by the sustained attention task from the determined $J$ components. Different methods of selecting task-modulated TCA components have been described in previous studies such as a method integrating the prior knowledge of multi-domain components with a significant difference between experimental conditions (Cong et al. 2013b), a method combining the prior knowledge with significant correlations between variations of components and musical features (Zhu et al. 2019), and a method matching prior knowledge and significant task-modulation FC (empirical null distribution constructed based on phase randomization) (Zhu et al. 2020a). In the present study, we performed the selection procedure based on the prior knowledge of temporal windows, frequency bands, and brain networks involved in sustained attention tasks and the significant correlations between representations of components and behavioral data.

On the one hand, prior knowledge has revealed that implanting a sustained attention task involves different neural functions such as attentional preparatory, attentional stability, working memory, and enhancement/inhibition of selected/ unselected information (Clark et al. 2015; Reteig et al. 2019; Rosenberg et al. 2016; Slagter et al. 2016). Regarding the multi-domain TCA components, the FC patterns at different frequency bands should emerge in different time windows to subserve a variety of functions in sustained attention. Previous studies have demonstrated that preparatory orienting of attention is indexed by the pre-stimulus alpha, activated in the right-lateralized visual hemifield (Reteig et al. 2019; Worden et al. 2000). The variability of attention processing has been associated with the post-stimulus theta phase coherence, activated in frontal and parieto-occipital brain regions (Lutz et al. 2009; Reteig et al. 2019). The FC between frontal, parietal, and occipital brain regions in delta and theta bands has been linked to the working memory (Düzel et al. 2010; Gulbinaite et al. 2014; Harper et al. 2017). Numerous research work has demonstrated that the beta power (13–30 Hz) in the motor cortex is related to movement execution and response inhibition (Pfurtscheller and Aranibar 1977; Pfurtscheller and Lopes Da Silva 1999; Zabielska-Mendyk et al. 2018). The 20 Hz (mu) rhythm in particular is associated with the motor cortical function of the hand, and even unimanual finger movement relates to the bilateral somatomotor cortex (Hari and Salmelin 1997).

**Fig. 3:**
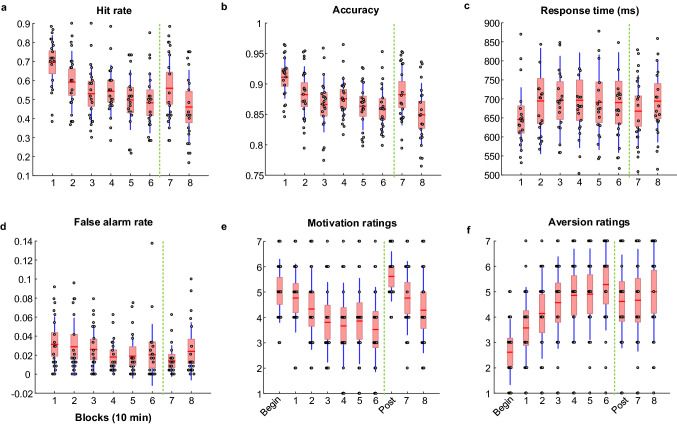
The changes in behavioral measurements and questionnaire ratings affected by time-on-task and motivation. **a** Hit rate and **b** Accuracy declined with time-on-task and transiently recovered (block 7) after providing rewards (after 60 min, as marked with the vertical dotted green line). **c** Response time decreased with time-on-task and showed a partial restoration pattern in block 7 although no significant difference. **d** False alarm rate did not change with time-on-task. **e** Motivation ratings and **f** Aversion ratings were influenced by time-on-task, and the motivation ratings restored to the initial level whereas the aversion ratings remained high compared to the initial level. The ratings of motivation and aversion were conducted before the task performance (begin) and after providing the reward instructions (post), as well as every 10 min task performance. The red line in the box represents the mean value, the light red box represents the standard deviation (SD), and the blue line corresponds to the 95% confidence interval. Note: The statistical results of the behavioral measurements and questionnaire ratings have been presented (Reteig et al. 2019). We only displayed the changes of them with scatter and boxplot to directly reveal the reliability for correlation analysis to select the TCA components

On the other hand, behavioral measurements—hit rate, a variant of accuracy, and response time—and motivation and aversion ratings have been illustrated as reliable markers, reflecting the effects of time-on-task and motivation (Reteig et al. 2019). The changes in behavioral and questionnaire data are shown in Fig. 3. The hit rate, accuracy, and response time rather than the false alarm rate deteriorated with time-on-task and transiently restored by motivation. Consequently, these three behavioral measurements were used for association analysis to select task-modulated TCA components. Although the ratings of motivation and aversion were significantly modulated by vigilance decrement and motivation, they were not applied for component selection because these ratings were not correctly corresponding to the values of each block.

We conducted correlation analyses between behavioral measurements and features of TCA components by connecting these measurements and features from all blocks (a total of 168 samples in the correct rejection condition and 84 samples in the hits and misses conditions). We selected the desirable TCA components that both consistent with prior knowledge with a visual inspection and closely related to the behavioral measurements. The component which only meets the criterion of prior knowledge or only meets the criterion of significant correlations were not selected, as shown in Figs. S1–3.

### Statistics

In order to examine the changes of fdFC caused by vigilance decrement, the elements in feature factor were subjected to the one-way analysis of variances (ANOVAs) with the within-subject factor block (first 60 min without reward) and results were corrected by the Greenhouse–Geisser. We also conducted the pair-wise comparisons between blocks 6 and 1 in the correct rejections condition and between blocks 3 and 1 in the hits and misses conditions to directly reveal the differences of fdFC between low and high vigilance state (without considering variations in different blocks). We performed the pair-wise comparisons between blocks 7 and 6 in the correct rejections condition and between blocks 4 and 3 in the hits and misses conditions to indicate the modulations of fdFC produced by motivation. When the effects of reward on specific component were detected, we ran pair-wise comparisons between blocks 7 and 1 in the correct rejections condition and between blocks 4 and 1 in the hits and misses conditions to quantitatively investigate the improvement of fdFC by motivation relative to that in the high vigilance state. In case of significant differences between blocks 7 and 1, we further ran the comparisons between blocks 8 and 1 to explore the continuous effect of motivation on fdFC. Both paired-sample t-test and Kruskal–Wallis test were applied to pair-wise comparisons. When the representations of the specific component followed the Gaussian distribution measured by the Jarque–Bera test, we used the paired-sample t-test, otherwise the Kruskal–Wallis test.

After testing the distribution of behavioral data and representations of component with Jarque–Bera test, the Pearson correlation (following the Gaussian distribution) or Spearman rank correlation (non-parametric test) were used for correlation analysis between behavioral measurements (e.g., hit rate, accuracy, and response time) and the features of TCA components.

The type I errors should be controlled during multiple comparisons when more than one TCA component was selected and multiple pair-wise comparisons were conducted. The p-values from selected components were corrected by the false discovery rate (FDR) to control the false discoveries (Benjamini and Yekutieli 2005, 2001; Benjamini and Hochberg 1995). The p-values (under one-tailed condition) from multiple pair-wise comparisons were further corrected. In sum, the statistics were conducted in MATLAB 2018b and IBM SPSS Statistics version 22. All tests applied a significance level of $0.05$.

## Results

The number of TCA components were determined by the DIFFIT, and the components modulated by the sustained attention task were selected from the retained TCA components using the criterion of prior knowledge and the significant correlations with behavioral measurements. We presented the multi-domain TCA components involved in the sustained attention task in the conditions of correct rejections, hits, and misses, and showed the variations of fdFC affected by vigilance decrement and motivation.

### TCA Components in the Correct Rejections Condition

**Fig. 4:**
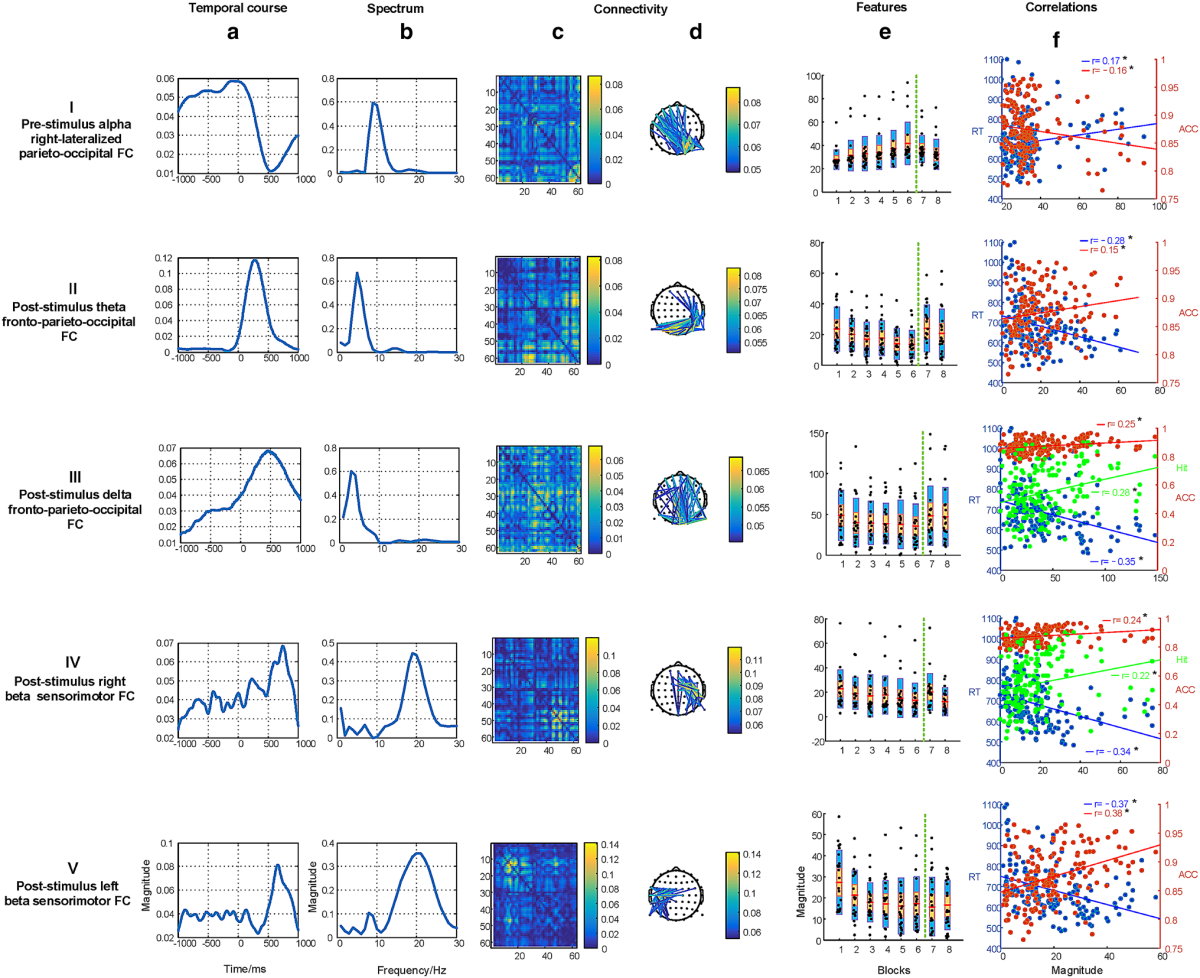
The selected five TCA components in the correct rejections condition. Each row represents one component, consisting of four dimensional information: the temporal factor showing the temporal course during sustained attention (**a**); the spectral factor showing the involvement of specific spectrum (**b**); the connectivity factor representing the symmetrical weighted FC matrix (**c**) and the 2D weighted connectivity visualization (showing the top 2% of the links with highest values, and the 2% thresholding was only used for visualization), with different colors related to different connectivity strengths (**d**); the features of fdFC affected by time-on-task (blocks 1–6, marked with the vertical dotted green line) and motivation (blocks 7 and 8), and the red line in the bar represents the mean value, the yellow bar represents the standard deviation (SD), and the blue bar corresponds to the 95% confidence interval (Loftus and Masson 1994) (**e**). Correlations between behavioral measurements, namely the response time (RT, in blue line), accuracy (ACC, in red line), and Hit rate (Hit, in green line) and variations of blocks across all subjects are displayed, significant relationships (p < 0.05) marked with * (**f**). Note that the insignificant correlations between the hit rate and features are not presented in scatters but in Fig. S1

According to the DIFFIT, a total of 25 TCA components were determined in the inhibition of the correct rejections condition. Among the 25 components, we selected 5 task-modulated components using the criterion based on prior knowledge and significant associations between representations of components and behavioral data. The five task components could be considered four types of neuromakers: (I) the pre-stimulus alpha right-lateralized parieto-occipital FC, (II) the post-stimulus theta fronto-parieto-occipital FC, (III) the post-stimulus delta fronto-parieto-occipital FC, (IV) the post-stimulus beta right sensorimotor FC, and (V) the post-stimulus beta left sensorimotor FC, as shown in Fig. 4.

#### Pre-stimulus Alpha Parieto-Occipital FC

The component as shown in row I of Fig. 4 had significant associations with the response time ($r=0.17, p=0.03$) and accuracy ($r=-0.16, p=0.04$). The FC that activated in the parietal and occipital brain regions, was right-lateralized. This FC emerged in the time window of − 1000 to 0 ms stimulus-onset was dominated by the alpha band. The strength of the fdFC was affected by vigilance decrement, with a slight increase with time-on-task (${F\left(3.07, 61.31\right)=2.86, p}_{corr}=0.057$). Moreover, the strength was stronger in block 6 than in block 1 (${t\left(20\right)=2.47, p}_{corr}=0.024$). There was no significant difference between blocks 7 and 6, although the strength of the fdFC showed a decrease pattern (${t\left(20\right)=1.22, p}_{corr}=0.149$). Neither a significant difference between blocks 1 and 7 (${t\left(20\right)=1.57, p}_{corr}=0.124$) nor between blocks 1 and 8 (${t\left(20\right)=1.08, p}_{corr}=0.184$) was detected.

#### Post-stimulus Theta Fronto-Parieto-Occipital FC

The component in Row II of Fig. 4 was significantly correlated with the response time ($r=-0.28, p<0.01$) and accuracy ($r=0.15, p=0.05$). The temporal window of the fronto-parieto-occipital FC ranged from 100 to 500 ms stimulus-onset, and the spectrum of it spanned the theta band. The strength of the fdFC decreased slightly during a long period of task engagement (${F\left(3.47, 69.41\right)=2.61, p}_{corr}=0.057$). The strength was weaker in block 6 than in block 1 (${t\left(20\right)=2.32, p}_{corr}=0.024$). Monetary reward increased the strength in block 7 relative to block 6 (${t\left(20\right)=2.59, p}_{corr}=0.022$). The strength of the fdFC in block 1 was comparable to that in block 7 (${t\left(20\right)=0.57, p}_{corr}=0.358$) and block 8 (${t\left(20\right)=1.11, p}_{corr}=0.184$).

#### Post-stimulus Delta Fronto-Parieto-Occipital FC

The component (row III of Fig. 4) was positively associated with the hit rate ($r=0.28, p<0.01$) and accuracy ($r=0.25, p<0.01$), and negatively associated with the response time ($r=-0.35, p<0.01$). The fronto-parieto-occipital FC peaked around 460 ms in the temporal course with the spectral modes ranging from 1–4 Hz corresponding to the delta band. There was a slight deterioration of the fdFC strength during a prolonged duration of task involvement (${F\left(3.83, 76.61\right)=2.01, p}_{corr}=0.092$). The strength was weaker in block 6 than in block 1 (${t\left(20\right)=2.26, p}_{corr}=0.024$). The strength of the fdFC in block 7 increased relative to block 6 after providing rewards (${t\left(20\right)=2.78, p}_{corr}=0.022$). There was no significant difference either between blocks 1 and 7 (${t\left(20\right)=0.01, p}_{corr}=0.500$) or between blocks 1 and 8 (${t\left(20\right)=0.28, p}_{corr}=0.392$).

#### Post-stimulus Right and Left Beta Sensorimotor FCs

The component (row IV of Fig. 4) had a positive relationship with the hit rate ($r=0.22, p<0.01$) and accuracy ($r=0.24, p<0.01$), and had a negative relationship with the response time ($r=-0.34, p<0.01$). The right-lateralized sensorimotor FC that peaked around 740 ms stimulus onset in the temporal course and was dominated by 20 Hz in the spectrum. The strength of the fdFC was weaker in block 6 than in block 1 (${t\left(20\right)=2.07, p}_{corr}=0.026$) and it was stronger in block 7 than in block 6 after providing rewards (${t\left(20\right)=2.30, p}_{corr}=0.033$). There was no significant difference between blocks 1 and 7 (${t\left(20\right)=0.86, p}_{corr}=0.333$), whereas the strength of this fdFC was weaker in block 8 than in block 1 (${t\left(20\right)=2.35, p}_{corr}=0.044$).

The component (row V of Fig. 4) was closely related to the response time ($r=-0.37, p<0.01$) and accuracy ($r=0.38, p<0.01$). The left-lateralized sensorimotor FC that peaked around 670 ms stimulus onset was dominated by 20 Hz in the spectrum. A slight deterioration of the left sensorimotor FC was detected with time-on-task (${F\left(2.32, 46.36\right)=2.53, p}_{corr}=0.057$). The strength of it was weaker in block 6 than in block 1 (${t\left(20\right)=2.21, p}_{corr}=0.024$). There was no improvement of the strength in block 7 after manipulating motivation (${t\left(20\right)=0.46, p}_{corr}=0.326$).

### TCA Components in the Hits Condition

**Fig. 5:**
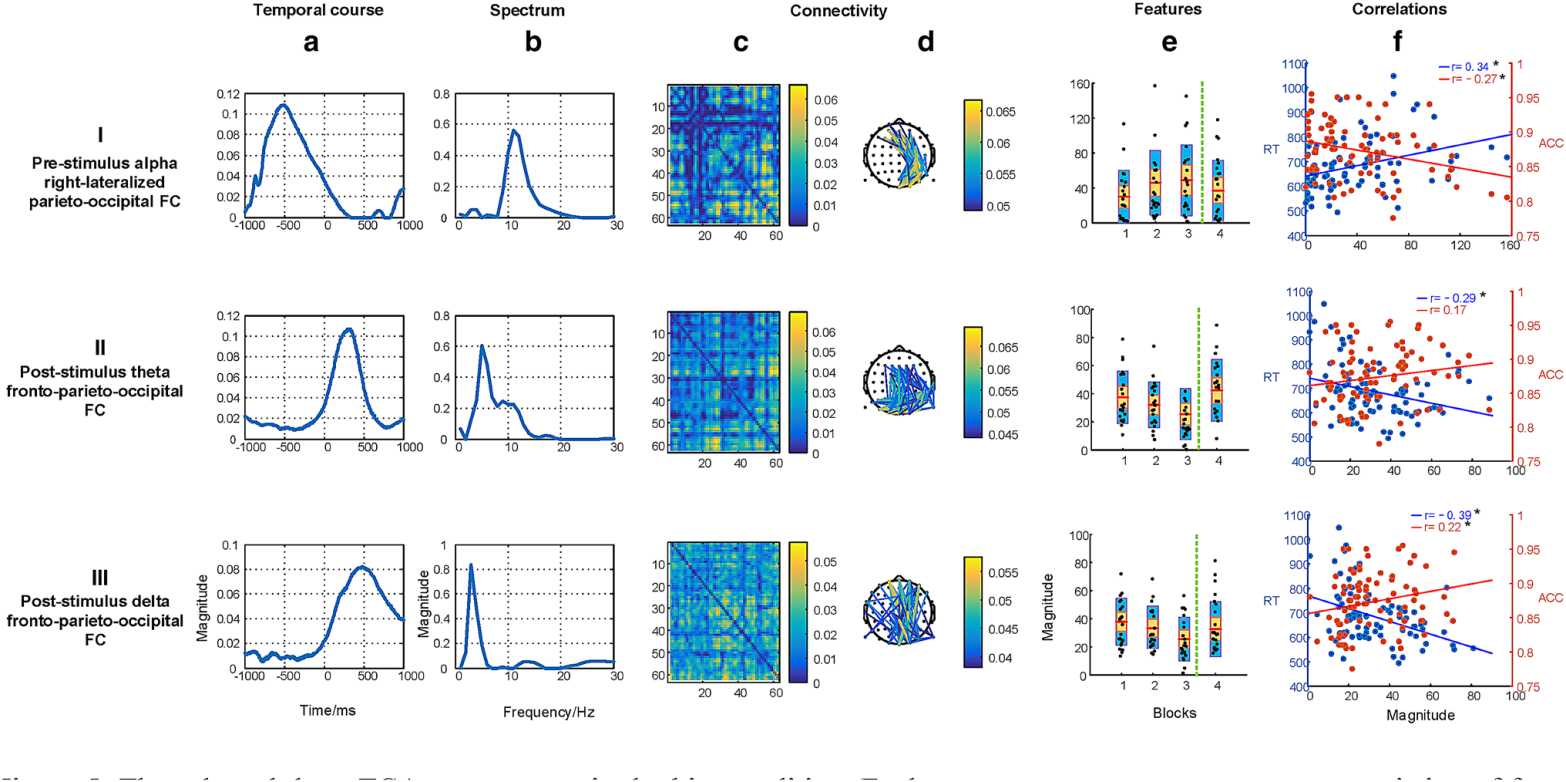
The selected three TCA components in the hits condition. Each row represents one component, consisting of four dimensional information including the temporal factor showing time varies during sustained attention (**a**), the spectral factor showing the specific oscillatory activations in the corresponding FC (**b**), the connectivity factor representing the symmetrical weighted connectivity matrix (**c**) and the 2D weighted connectivity visualization (showing the top 2% of the links with highest values, and the thresholding was only used for visualization), with different colors related to different connectivity strengths (**d**), and features’ changes affected by time-on-task (blocks 1–3, marked with the vertical dotted green line) and motivation (block 4), and the red line in the bar represents the mean value, the yellow bar represents the standard deviation (SD), and the blue bar corresponds to the 95% confidence interval (**e**). Correlations between behavioral measurements, namely the response time (RT), and accuracy (ACC), and features are presented, with significant relationships (p < 0.05) marked with * (**f**). Note that the insignificant correlations between the hit rate and features are not presented in scatters but in Fig. S2

In the condition of hits, we extracted 35 TCA components based on the DIFFIT criterion and finally selected 3 task-modulated components according to the mentioned criterions. We derived three neuromarkers including the pre-stimulus alpha right-lateralized parieto-occipital FC (row I of Fig. 5), the post-stimulus theta fronto-parieto-occipital FC (row II of Fig. 5), and the post-stimulus delta fronto-parieto-occipital FC (row III of Fig. 5). These three neuromarkers were also discovered in the correct rejections condition. However, the beta right/left sensorimotor FCs were not detected in the hits condition.

#### Pre-stimulus Alpha Parieto-Occipital FC

The component as shown in row I of Fig. 5 was significantly correlated with the response time ($r=0.34, p<0.01$) and accuracy ($r=-0.27, p=0.01$). The FC was mainly activated in the right-lateralized parietal and occipital brain regions. The FC emerged in the time window of –1000 to 0 ms was dominated by the alpha band. The strength of the fdFC increased during the sustained attention task over time (${F\left(1.91, 36.29\right)=5.32, p}_{corr}=0.027$) and the strength was stronger in block 3 than in block 1 (${t\left(20\right)=-3.43, p}_{corr}=0.003$). After providing rewards, the strength in block 4 decreased compared to block 3 (${t\left(20\right)=3.43, p}_{corr}=0.004$) and the strength in block 4 recovered to the initial level in block 1 (${t\left(20\right)=0.58, p}_{corr}=0.284$).

#### Post-stimulus Theta Fronto-Parieto-Occipital FC

There was a close association between the component (row II of Fig. 5) and the response time ($r=-0.29, p=0.01$). The temporal course of the fronto-parieto-occipital FC ranged from 0 to 500 ms and the spectrum of it peaked around 5 Hz. There was a decline of the strength of the fdFC during prolonged task engagement (${F\left(1.57, 31.35\right)=3.26, p}_{corr}=0.049$). The strength was weaker in block 3 than in block 1 (${t\left(20\right)=3.38, p}_{corr}=0.003$). The strength in block 4 was improved after providing incentives compared to block 3 (${t\left(20\right)=2.61, p}_{corr}=0.013$). This improvement reached the strength of it in block 1, with no difference between blocks 1 and 4 (${t\left(20\right)=0.95, p}_{corr}=0.285$).

#### Post-stimulus Delta Fronto-Parieto-Occipital FC

The component (Row III of Fig. 5) was significantly associated with the response time ($r=-0.39, p<0.01$) and accuracy ($r=0.22, p=0.47$). The fronto-parieto-occipital FC that peaked around 490 ms in temporal course was dominated by the delta band. Results revealed a decline of the strength of the fdFC with time-on-task (${F\left(1.69, 33.75\right)=3.65, p}_{corr}=0.049$). The strength was weaker in block 3 than in block 1(${t\left(20\right)=2.76, p}_{corr}=0.012$). A slight increase of the strength in block 4 was detected after providing incentives compared to block 3 (${t\left(20\right)=1.70, p}_{corr}=0.053$). There was no significant difference between blocks 1 and 4 (${t\left(20\right)=0.45, p}_{corr}=0.467$).

### TCA Components in the Misses Condition

**Fig. 6:**
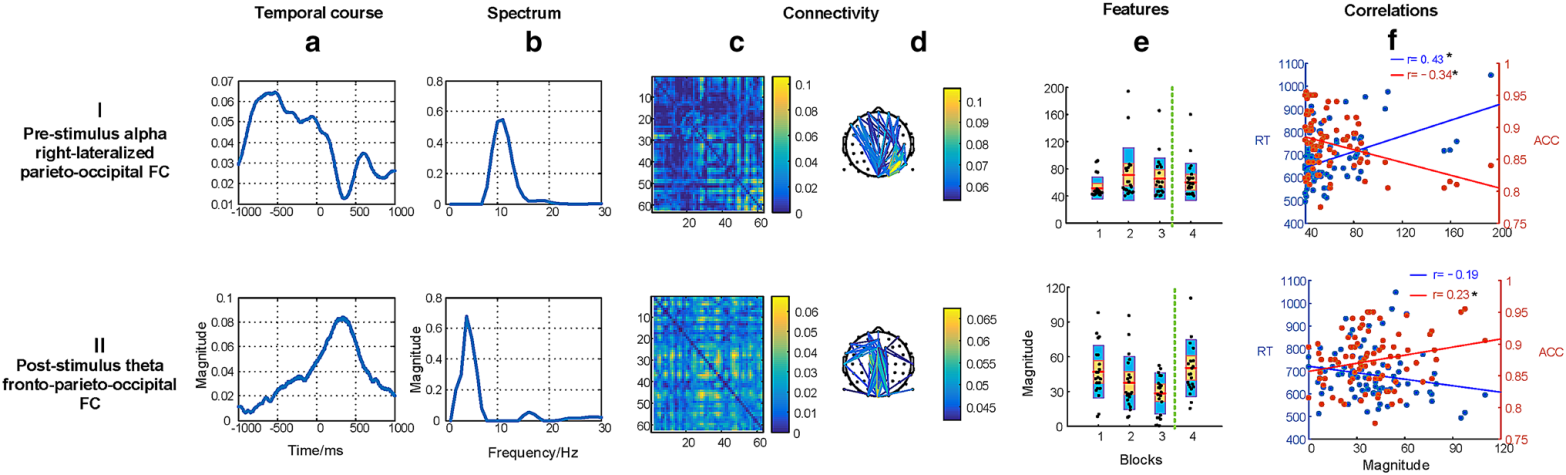
Two selected TCA components in the misses condition. Each row represents one component, consisting of the temporal factor showing the time varies (**a**); the spectral factor showing the dominated frequency band (**b**); the connectivity factor representing the symmetrical weighted connectivity matrix (**c**) and the 2D weighted connectivity visualization (showing the top 2% of the links with highest values, and the thresholding was only used for visualization), with different colors related to different connectivity strengths (**d**); the features’ factor affected by time-on-task (blocks 1–3, marked with the vertical dotted green line) and motivation (block 4), and the red line in the bar represents the mean value, the yellow bar represents the standard deviation (SD), and the blue bar corresponds to the 95% confidence interval (**e**). Correlations between behavioral measurements, namely the response time (RT, in blue line), and accuracy (ACC, in red line), and variations of blocks across all subjects are displayed, significant relationships (p < 0.05) marked with * (**f**). Note that the insignificant correlations between the hit rate and features are not presented in scatters but in Fig. S3

We extracted 25 TCA components based on the DIFFIT and finally selected two components modulated by the sustained attention task, representing (I) the pre-stimulus alpha right-lateralized parieto-occipital FC and (II) the post-stimulus theta fronto-parieto-occipital FC, as shown in Fig. 6. Similar to the hits condition, the beta right/left sensorimotor FCs were not detected in the misses condition, whereas different from the hits condition, the delta fronto-parieto-occipital FC was not observed in the misses condition.

#### Pre-stimulus Alpha Parieto-Occipital FC

The component in row I of Fig. 6 was positively correlated with the response time ($r=0.43, p<0.01$) and negatively correlated with the accuracy ($r=-0.34, p<0.01$). The right-lateralized parieto-occipital FC emerged mainly around − 1000 to 0 ms stimulus-onset and was dominated by the alpha band. The strength of the fdFC increased slightly with time-on-task (${F\left(1.95, 39.04\right)=3.38, p}_{corr}=0.068$) and the strength was stronger in block 3 than in block 1 (${t\left(20\right)=2.46, p}_{corr}=0.023$). The impaired strength was not modulated by rewards, with no difference between blocks 4 and 3 (${t\left(20\right)=1.26, p}_{corr}=0.112$).

#### Post-stimulus Theta Fronto-Parieto-Occipital FC

Row II of Fig. 6 shows a component that has a significant relationship with the accuracy ($r=0.23, p=0.039$). The fronto-parieto-occipital FC ranged in the time window of 0–500 ms and peaked around 5 Hz in the spectrum. There was a slight decline of the strength of the fdFC with time-on-task (${F\left(1.65, 32.89\right)=2.83, p}_{corr}=0.068$). The strength was weaker in block 3 than in block 1 (${t\left(20\right)=2.85, p}_{corr}=0.020$). The strength in block 4 increased relative to block 3 (${t\left(20\right)=2.90, p}_{cor}=0.009$) after providing rewards and the increment of the strength reached the level of it in block 1 (${t\left(20\right)=-0.55, p}_{corr}=0.443$).

## Discussion

By applying the analysis framework composed of wPLI and TCA to the high-temporal resolution EEG collected during a sustained attention task over 80 min, we examined how a cascade of fundamental functions was reflected by the fdFC and which stages or a combination of stages were affected by vigilance decrement and motivation. In tandem, we performed the analysis framework in the correct rejections, hits, and misses conditions to explore the distinctive involvement of functions in different conditions. Following the main steps of the framework (Fig. 2), we firstly calculated the time–frequency domain wPLI from whole-brain electrodes in each block and subject and then constructed a fourth-order tensor (Time × Frequency × Connectivity × (Subjects × Blocks)) by concatenating the data in blocks and subjects. Afterward, the TCA was applied to the fourth-order tensor to characterize the interacted, low dimensional, and representative components, suggesting the when (specific time windows), how (particular frequency band), and where (definite brain regions) of sustained attention were affected by vigilance decrement. A total of four types of neuromarkers were identified, namely the pre-stimulus alpha right-lateralized parieto-occipital FC, the post-stimulus theta fronto-parieto-occipital FC, delta fronto-parieto-occipital FC, and beta right/left sensorimotor FCs. These fdFCs emerged in different time windows and conditions to support the implementation of a sustained attention task. Results demonstrated that all fdFCs were impaired by vigilance decrement, but they were differently modulated by motivation. The pre-stimulus alpha parieto-occipital FC and the post-stimulus theta and delta fronto-parieto-occipital FCs were restored by the motivation to the initial level, but the beta left sensorimotor FC was not modulated by motivation. Interestingly, the beta right sensorimotor FC increased only in the first 10 min but decreased in the last 10 min during the interval of motivation manipulation. Taken together, assisted with the tensor-based framework, we successfully derive a sequence of fdFCs involved in sustained attention and the discrepancies of these fdFCs among distinct conditions, as well as the organizations of them modulated by time-on-task and motivation.

### Horizontal Analysis: Fundamental Functions in Sustained Attention

In the present study, the time window of the alpha right-lateralized parieto-occipital FC ranged from − 1000 to 0 ms. The theta fronto-parieto-occipital FC peaked around 280–320 ms and the delta fronto-parieto-occipital FC peaked around 460–490 ms across emerged conditions. The beta (peaked at approximately 20 Hz) right and left sensorimotor FCs peaked around 670 and 740 ms, respectively. These fdFCs emerged in the temporal order of the alpha parieto-occipital, theta fronto-parieto-occipital, delta fronto-parieto-occipital, and beta right/left FCs. When these results are interpreted in the context of the roles suggested for the fdFCs, a series of fundamental functions underlying sustained attention can be tracked.

A previous study has demonstrated that the event-related desynchronization within the alpha band in the occipital brain regions is associated with the anticipatory attention for a forthcoming stimulus (Bastiaansen et al. 2001). Moreover, the lateralization of alpha is a critical index of spatial attention (Thut et al. 2006). A large body of research has shown that the alpha band increases in the ipsilateral hemisphere while decreases in the contralateral hemisphere when humans deploy their attention to one location (Thut et al. 2006; Worden et al. 2000). The alpha right-lateralized parieto-occipital FC in our study might be an index of anticipatory attention, although the lateralization is opposite to that in earlier studies. These diverging results concerning the lateralization might result from the specific task design where the stimulus was presented only on the left of the fixation, as pointed out in an earlier publication based on the same dataset (Reteig et al. 2019). Secondly, the post-stimulus theta phase coherence in fronto-parieto-occipital topography has been linked to the attentional stability (Lutz et al. 2009) and the variability of brain responses (Reteig et al. 2019). In line with these previous studies, our results also observed the involvement of the frontal, parietal, and occipital brain regions in the theta band, suggesting that the attentional stability might be indexed by the theta fronto-parieto-occipital FC. Next, earlier work has suggested that the fronto-occipital brain network in delta band is closely related to the working memory (Gulbinaite et al. 2014; Harper et al. 2017). Consistent with these findings, the delta fronto-parieto-occipital FC extracted in our study is likely to relate to the working memory. Finally, the close relationship between the beta band and the response movement has been built in the literature (Pfurtscheller and Aranibar 1977). The 20 Hz mu rhythm is particularly associated with the motor cortical function, with the bilateral engagement of the somatomotor cortex even in unilateral movement (Hari and Salmelin 1997). In the present study, the beta (peaked around 20 Hz) right/left somatomotor FCs might provide evidence for response movement.

In sum, according to the timeline and the cognitive content, the four types of neuromarkers appear to correspond to a cascade of fundamental functions in sustained attention consisting of the attentional preparatory, attentional stability, working memory, and response movement.

### Vertical Analysis: Functional Discrepancies in Different Conditions

Our study detected the pre-stimulus alpha parieto-occipital FC and the post-stimulus theta fronto-parieto-occipital FC in the correct rejections, hits, and misses conditions. The alpha and theta FCs continuously presented despite the different responses participants conducted. In line with our findings, a different set of results have reported that the pre-stimulus alpha power and the post-stimulus theta phase presented in these three conditions (Reteig et al. 2019). Integrating the fdFCs with the fundamental functions underlying sustained attention, these findings appear to indicate that people need to prepare attention for each upcoming stimulus and the attentional stability exists in all three conditions.

The delta fronto-parieto-occipital FC was derived only in correct responses, including correctly inhibiting non-target (the correct rejections condition) and detecting target (the hits condition), but not in error responses of detecting target (the misses condition). As referred above, the delta FC is related to working memory. It is likely that the inability to detect targets is owing to a failure of the target-related working memory process. The working memory encompasses subprocesses of information encoding, maintenance, or retrieval (Düzel et al. 2010; Quentin et al. 2019), but our results cannot infer which subprocess or a combination of them are dysfunctional in the misses condition.

A substantial amount of studies have examined the role of beta band in the motor cortex during reaction responses, suggesting that the response execution is related to beta rhythmic desynchronization and the response inhibition is associated with beta synchronization (Pfurtscheller and Aranibar 1977; Pfurtscheller and Lopes Da Silva 1999; Zabielska-Mendyk et al. 2018). The increased beta band during response inhibition to distractors is also well known as the “beta rebound” phenomenon (Bola and Sabel 2015). In this work, the beta right/left sensorimotor FCs emerged only in the correct rejections condition. Our findings are agreement with previous studies showing the synchronization of beta band in response inhibition and desynchronization in response execution. Interestingly, we did not extract the beta FCs in the misses condition although no responses were conducted in this condition. It is plausible that the inability to detect target is not the failure of response execution, but the failure of other functions such as working memory, which is consistent with the results presented by the delta fronto-parieto-occipital FC.

Unlike the magnitude differences—alpha power, N1/P1 component, and theta phase—between hits and misses conditions reported in the previous study (Reteig et al. 2019), the present work successfully found the functional discrepancies in the hits, misses, and correct rejections conditions. The patterns of fdFC shift to subserve distinct responses (e.g., correct rejections, hits, misses) during sustained attention.

### The fdFCs Affected by Vigilance Decrement and Motivation

The pre-stimulus alpha parieto-occipital FC was more right-lateralized with the decrease of vigilance and less right-lateralized after manipulating motivation in our study. Similar results have been reported in an earlier study that increased attentional load and time-on-task give rise to more right-lateralization in posterior alpha asymmetry (Newman et al. 2013), although the earlier study does not explore the changes of lateralization influenced by motivation. Our results reaffirm that the non-spatial factor of time-on-task modulates the biases of spatial attention and further verify that the motivation is another non-spatial factor influencing the attention biases. In contrast to most externally-cued attention orienting studies, our study and the earlier study (Newman et al. 2013) did not set up pre-target cues, but we demonstrated that the anticipatory pre-stimulus alpha was also apparent in no-cued attention orienting.

We found that the post-stimulus theta fronto-parieto-occipital FC decreased with time-on-task and increased after manipulating motivation. In line with previous studies (Lutz et al. 2009; Reteig et al. 2019), the theta FC could be interpreted as a reliable index indicating the changes of attentional stability or brain responses modulated by time-on-task and motivation. These results suggest that human attentional stability could be impaired by vigilance decrement and this ability could be restored after providing rewards.

The post-stimulus delta fronto-parieto-occipital FC decreased with time-on-task and increased after providing rewards, indicating that the function of working memory in sustained attention was impaired by vigilance decrement and recovered by rewards. The impairment by the decrement of vigilance is supported by a piece of indirect evidence that higher working memory capacity is related to weaker fronto-parietal FC (Gulbinaite et al. 2014). Consistent with our results, a previous study has demonstrated the enhancement effect of reward on the working memory capacity (Sanada et al. 2013). Our finding seems to show that the working memory, at least the function involved in sustained attention, is sensitive to both time-on-task and motivation.

The post-stimulus beta right/left sensorimotor FCs decreased after long durations of task performance. Akin to a previous study (Guo et al. 2018), we elucidated that time-on-task is one of the main factors leading to the degrading of response inhibition. We also found that the ipsilateral right sensorimotor FC, but not the contralateral left sensorimotor FC, was restored by motivation when participants inhibited with a right finger’s response. This appears to illustrate that the right sensorimotor activation is more sensitive to motivation than the left sensorimotor, in the situation that bilateral somatomotor networks are engaged in the unilateral movement. Although we cannot make conclusions on the source of the reward restoration, the connections between the right hemisphere and response inhibition have also been built in an earlier research (Aron et al. 2003). Another theoretical work also suggests that successful versus unsuccessful inhibited differential responses are related to the right hemisphere during rewarded condition (Padmala and Pessoa 2010).

### Transient and Partial Restoration of fdFCs After Manipulating Motivation

Prolonged performance (60 min) of a sustained attention task lead to vigilance decrement, impairing all types of fdFCs. Participants were motivated with an extra monetary reward in the last 20 min interval. Four types of fdFCs, except for the left sensorimotor FC, were restored by reward, although the recovery of the right sensorimotor FC was transient. The right sensorimotor FC increased only in the first 10 min and then fell down to the low vigilance level in the last 10 min. The restoration of fdFCs by motivation appears to inconsistent with the overload theories, which states that cognitive resources are limited and vigilance decrement is determined only by resource depletion. The partial and transient improvement by motivation might agree with the motivational control and energetical costs theoretical model, where participants evaluate the costs and benefits and also assess their energetic resources to decide to expend or reserve their efforts.

There are still three caveats in explaining the results. First of all, the modulation effect of reward on the pre-stimulus alpha parieto-occipital FC is detected in the correct rejections and hits conditions, but not in the misses condition. It is possible that the differences are from the robustness of motivation itself or the instability of preparation of attention in the misses condition. Secondly, the number of trials cannot be equalized in the correction rejections and hits/miss conditions because of the experimental design. This imbalance might result in some differences in the TCA components. Finally, the task-related component selection is critical for the application of the tensor-based framework. Not only with the behavioral data association analyses but also more robustness and efficient methods should be developed for TCA component selection to provide comprehensive consideration.

## Conclusion

We apply an analysis framework composed of wPLI and TCA to a long period of sustained attention EEG dataset and derive a cascade of fdFCs involved in a sustained attention task. The pre-stimulus alpha parieto-occipital FC, post-stimulus theta fronto-parieto-occipital FC, delta fronto-parieto-occipital FC, and beta right/left sensorimotor FCs are derived, corresponding to different functions in sustained attention. We successfully detect the modulations of fdFCs affected by vigilance decrement and motivation. All these fdFCs are impaired by vigilance decrement. Especially, the pre-stimulus alpha FC parieto-occipital drifts rightward with time-on-task. The impairments of fdFCs are partially restored by motivation. The post-stimulus beta left sensorimotor network is not modulated by rewards. The right sensorimotor FC is more associated with motivation than the left sensorimotor FC, although the effect of improvement by motivation on the right sensorimotor FC is transient. Our results lay the ground for the hybrid model that vigilance decrement is determined by motivational control and energetical costs. The analysis framework provides feasibility for identifying dynamic organizations of frequency-specific FC in cognitive tasks.

## Electronic Supplementary Material

Below is the link to the electronic supplementary material.

Supplementary file 1 (DOCX 19053 kb)
