# Supplementary material for: Sustaining Attention for a Prolonged Duration Affects Dynamic Organizations of Frequency-Specific Functional Connectivity
Source: Brain Topogr. 2020 Sep 14;33(6):677–92. doi: 10.1007/s10548-020-00795-0 (PMC7593315; doi:10.1007/s10548-020-00795-0)
Supplement: Supplementary file 1 — (DOCX 19053 kb) [file 10548_2020_795_MOESM1_ESM.docx]

Figure S1. A total of 25 TCA components were derived in the correct rejections condition. The 5 task-related components (7, 11, 14, 19, 21) were selected and highlighted with dotted red frames. The component 7, with activations in right-lateralized parietal and occipital brain regions, was emerged in time windows of –1000 to 0 ms stimulus-onset and dominated by the alpha band. The component 7 is likely to link with the brain functions of attentional preparatory. The component 11, with fronto-parieto-occipital functional connectivity (FC) ranged from 100 to 500 ms stimulus onset and spanned the theta band, is probably associated with the attentional stability. Components 14 and 19, presented in the inhibition of responses, are corresponding to the right and left sensorimotor FCs, separately. The component 14 peaked around 740 ms stimulus onset and around 20 Hz in the spectrum, whereas component 19 peaked around 670 ms stimulus onset with almost the same 20 Hz in the spectrum. The component 21 was activated in fronato-parieto-occipital brain regions, peaked around 460 ms in the temporal course and dominated by delta band. This component appears to suggest the brain function of working memory. Each component consists of six columns, consisting of the features, temporal course, spectrum, connectivity matrix, 2D connectivity visualization (showing the top 2% of the links with the largest values, and the 2% thresholding was only used for visualization), and correlations with the hit rate (Hit), response time (RT), and accuracy (Acc).

Figure S2. A total of 35 TCA components were derived in the hits condition. The 3 task-related components (12, 13, 26) were selected and highlighted with dotted red frames. The component 12, with fronto-parieto-occipital FC ranged from 100 to 500 ms stimulus onset and spanned the theta band, is probably associated with the attentional stability. The component 13, activated in fronato-parieto-occipital brain regions, peaked around 490 ms in the temporal course, covered the delta band, appears to suggest the brain function of working memory. The component 26 appears to link to the brain functions of attentional preparatory, with activations in right-lateralized parietal and occipital brain regions, emerged in time windows of –1000 to 0 ms stimulus-onset, and dominated by the alpha band. Each component consists of six columns, consisting of the features, temporal course, spectrum, connectivity matrix, 2D connectivity visualization (showing the top 2% of the links with largest values, and the 2% thresholding was only used for visualization), and correlations with the hit rate (Hit), response time (RT), and accuracy (Acc).

Figure S3. A total of 25 TCA components were derived in the misses condition, among which 2 task-modulated components (5, 17) were selected and highlighted with dotted red frames. The component 5, with fronto-parieto-occipital FC ranged from 100 to 500 ms stimulus onset and spanned the theta band, is probably associated with the attentional stability. The component 17 is likely related to the brain functions of attentional preparatory, with activations in right-lateralized parietal and occipital brain regions, emerged in time windows of –1000 to 0 ms stimulus-onset, and dominated by the alpha band. Each component consists of six columns, consisting of the features, temporal course, spectrum, connectivity matrix, 2D connectivity visualization (showing the top 2% of the links with largest values, and the 2% thresholding was only used for visualization), and correlations with the hit rate (Hit), response time (RT), and accuracy (Acc).
